# Extension of the growing season increases vegetation exposure to frost

**DOI:** 10.1038/s41467-017-02690-y

**Published:** 2018-01-30

**Authors:** Qiang Liu, Shilong Piao, Ivan A. Janssens, Yongshuo Fu, Shushi Peng, Xu Lian, Philippe Ciais, Ranga B. Myneni, Josep Peñuelas, Tao Wang

**Affiliations:** 1 Sino-French Institute for Earth System Science, College of Urban and Environmental Sciences, Peking University, Beijing, 100871 China; 2 Key Laboratory of Alpine Ecology and Biodiversity, Institute of Tibetan Plateau Research, Chinese Academy of Sciences, Beijing, 100085 China; 3 Center for Excellence in Tibetan Earth Science, Chinese Academy of Sciences, Beijing, 100085 China; 4 Department of Biology, University of Antwerp, Universiteitsplein 1, Wilrijk, 2610 Belgium; 5 College of Water Sciences, Beijing Normal University, Beijing, 100875 China; 6 Laboratoire des Sciences du Climat et de l’Environnement, CEA CNRS UVSQ, Gif-sur-Yvette, 91191 France; 7 Department of Earth and Environment, Boston University, 675 Commonwealth Avenue, Boston, MA 02215 USA; 8 CREAF, Cerdanyola del Valles, Barcelona, Catalonia 08193 Spain; 9 CSIC, Global Ecology Unit CREAF- CSIC-UAB, Bellaterra, Barcelona, Catalonia 08193 Spain

## Abstract

While climate warming reduces the occurrence of frost events, the warming-induced lengthening of the growing season of plants in the Northern Hemisphere may actually induce more frequent frost days during the growing season (GSFDs, days with minimum temperature < 0 °C). Direct evidence of this hypothesis, however, is limited. Here we investigate the change in the number of GSFDs at latitudes greater than 30° N using remotely-sensed and in situ phenological records and three minimum temperature (*T*_min_) data sets from 1982 to 2012. While decreased GSFDs are found in northern Siberia, the Tibetan Plateau, and northwestern North America (mainly in autumn), ~43% of the hemisphere, especially in Europe, experienced a significant increase in GSFDs between 1982 and 2012 (mainly during spring). Overall, regions with larger increases in growing season length exhibit larger increases in GSFDs. Climate warming thus reduces the total number of frost days per year, but GSFDs nonetheless increase in many areas.

## Abstract (web summary)

Plant growing season increases under a warming climate, but it is not known whether this will alter plant exposure to frost days. Here Liu et al. investigate trends in the Northern Hemisphere over 30 years and find increased exposure to frost days in regions that have longer growing seasons.

## Introduction

Frost events during the growing season can affect the structure and function of terrestrial ecosystems by inhibiting plant growth^1–4^, reducing carbon uptake^5,6^, and disturbing nutrient cycling^7,8^. For example, the 2007 one-week spring freeze in central and eastern United States was estimated to have reduced the production of winter wheat by 19%, peaches by 75%, apples by 67%, and pecans by 66%, causing over $2 billion in economic losses^9^. Autumn freezing events, however, may accelerate or induce senescence, thereby killing plant tissues before maturity or before completing nutrient resorption^8^, and may also result in late summer crop yield losses^10,11^. Therefore, a better characterization and understanding is needed of changes in the occurrence of frost during the growing season.

Warming tends to reduce the number of frost days per year but also lengthens the growing season in temperature-limited ecosystems, which can in turn increase the period during which photosynthetically active vegetation is exposed to frost. Previous studies have hypothesized that the number of frost days during the growing season (GSFDs) will increase in response to lengthening growing season^5,12–14^, but, to our knowledge, this hypothesis has not been tested. In situ and satellite observations in the Northern Hemisphere have indicated that longer growing season have accompanied the regional warming trends over the last 30 years^15,16^. The long record and global coverage of satellite greenness data and gridded climatic data sets allow the quantification of decadal changes and trends in GSFDs. We documented the number of GSFDs in the Northern Hemisphere (with latitude greater than 30° N) for 1982–2012 using satellite-derived phenology data and three gridded climatic data sets (CRU-NCEP, Princeton, and WFDEI) (see Methods). Frost days were defined as days with *T*_min_ < 0 °C^17,18^.

We found that regions with larger increases in the length of the growing season have increasing frost days in the last three decades, despite the warming trends. In details ~43% of the hemisphere, especially in Europe and in spring, experiences a significant increase in GSFD during the last 30 years. Decreased GSFDs mainly occur in northern Siberia, the Tibetan Plateau, and northwestern North America, and mainly in autumn.

## Results

### Spatial pattern of frost days during the past three decades

**Fig. 1:**
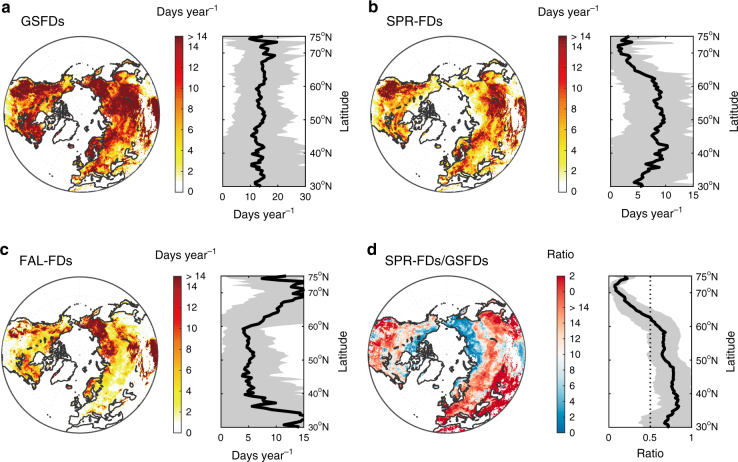
Spatial distribution of average frost days during growing season for 1982–2012. The number of frost days in the Northern Hemisphere was averaged from the results of the Princeton and WFDEI data sets. **a**–**c** indicates frost days and their variation along the gradient of latitude (black line and gray area presents the average frost days and its standard deviation across latitudes, respectively) calculated during growing season (GSFDs, from SOS to EOS), spring (SPR-FDs, from SOS to summer solstice) and autumn (FAL-FDs, from summer solstice to EOS). **d** displays the ratio of the number of frost days between (**b**) and (**a**). Maps were created using Matlab R2014b

Figure 1 shows the spatial distribution of the average number of GSFDs in the Northern Hemisphere during 1982–2012. This distribution is calculated using the 3-h Princeton and WFDEI temperature data sets by counting the number of days with *T*_min_ < 0 °C during the growing season (average of both data sets, see Methods). The largest numbers of GSFDs (>14 days per year) is found in western North America, northeast Europe, Siberia, and the Tibetan Plateau (Fig. 1a). The number of frost days in spring (SPR-FDs) vs. autumn (FAL-FDs) differs substantially among regions. FAL-FDs are more frequent in northeastern Siberia and the Tibetan Plateau (Fig. 1c), and SPR-FDs in western North America, northeastern Europe, and southeastern Siberia (Fig. 1b). The total number of GSFDs did not change with latitude (Fig. 1a), because the number of SPR-FDs (Fig. 1b) and FAL-FDs (Fig. 1c) show changes in opposite direction with increasing latitude. Figure 1d displays the fraction of the total number of frost days occurring in spring. The region south of 62 °N was mainly affected by SPR-FDs, but Arctic regions were dominated by FAL-FDs. Similar results were found using the middle day between SOS and EOS, instead of the summer solstice (Fig. 1), to separate SPR-FDs and FAL-FDs, suggesting that such spatial pattern of GSFDs at high latitudes was not due to a short spring (i.e., SOS close to summer solstice, Supplementary Fig. 1) (Supplementary Fig. 2a–d). The non-dominant role of SPR-FDs in GSFDs at high latitudes is possibly because most (>80%) of SPR-FDs occurred within a short time period (20 days) after the SOS (Supplementary Fig. 3a,b). The Princeton and WFDEI *T*_min_ observations provided consistent results (Supplementary Fig. 2i-p), and also the 6-h CRU-NCEP *T*_min_ observations indicated a similar, albeit weaker (i.e., fewer in the number), spatial pattern of GSFDs, probably because of its lower temporal resolution (Supplementary Fig. 2e–h).

### Change in the number of growing season frost days

**Fig. 2:**
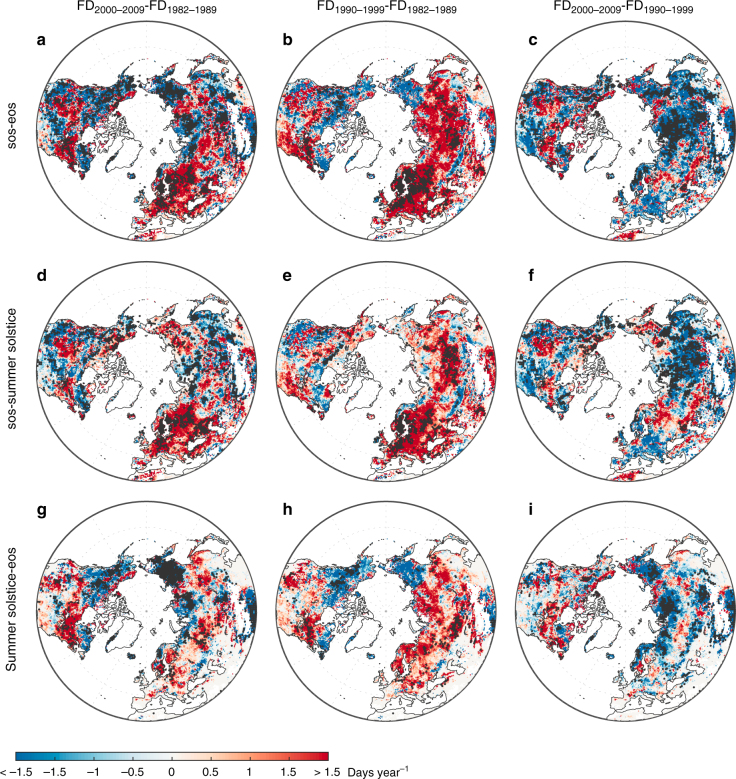
Decadal changes in average frost days during growing season. The number of frost days (FD) in the Northern Hemisphere was averaged from the results of the Princeton and WFDEI data sets. The left, center, and right panels indicate the differences in the average number of frost days between the 1980s and the 2000s, the 1980s and the 1990s, and the 1990s and the 2000s. The upper, middle, and bottom panels show the time periods used to calculate the number of frost days (**a**–**c** GSFDs, from SOS to EOS, **d**–**f** SPR-FDs, from SOS to the summer solstice, and **g**–**i** FAL-FDs, from the summer solstice to EOS). Dotted areas indicate regions with significant changes (*P* < 0.05, *t*-test) in the number of frost days. Maps were created using Matlab R2014b

We next examined the change in the number of GSFDs between the 1980s and the 2000s. During this period, mean northern growing-season temperatures (*T*_min_) increased by ~0.70 °C (0.75 °C in spring, from SOS to summer solstice, 0.67 °C in autumn, from summer solstice to EOS). Figure 2a shows the spatial distribution of changes in GSFDs. The number of GSFDs increased by more than one day per year over more than 36% of the Northern Hemisphere, mainly in Europe and central North America during the last 30 years. The temporal distribution of these additional GSFDs, however, differed between these two regions. The number of GSFDs increased over ~82% of the area of Europe, and the average increase in Europe was 2.8 ± 4.6 extra frost days per growing season (*P* < 0.05, *t*-test) (Fig. 2a). In Europe, increases occurred mostly in spring (2.7 ± 3.3 additional SPR-FDs per growing season, *P* < 0.05, *t*-test) (Fig. 2d). In contrast, GSFDs increased similarly in both spring and autumn in central North America (Fig. 2a, d, g). The increased SPR-FDs more likely occurred during short periods (i.e., 43% within 10 days and 81% within 1 month) after SOS (Supplementary Fig. 3c–f). GSFDs decreased in northern Siberia, the Tibetan Plateau, and northwestern North America (Fig. 2a), mainly due to a decrease in the number of FAL-FDs (Fig. 2g). Overall, GSFDs decreased significantly in about 34% of the Northern Hemisphere, which is slightly lower than the area (~43%) that experienced a significant increase (Fig. 2a). The percentage of land areas where GSFDs increased significantly was similar in autumn and spring (~40 versus ~45%) (Fig. 2d, g). We derived similar results when using the CRU-NCEP, Princeton, and WFDEI *T*_min_ data sets individually (Supplementary Fig. 4) instead of combining the Princeton and WFDEI data sets (Fig. 2).

Both growing season length (GSL) and temperature increased between the 1980s and the 1990s and showed no significant change between the 1990s and the 2000s^19–22^. The 2000s is marked by a warming hiatus in the Northern Hemisphere, with decreasing temperature in spring over North America and winter over Eurasia^23,24^. We examined the effects of changing GSL and *T*_min_ on GSFDs by analyzing the differences in the number of GSFDs between the 1980s and the 1990s and between the 1990s and the 2000s (Fig. 2b, c). The GSFDs increased across more than 66% of the Northern Hemisphere area between the 1980s and the 1990s (significant over ~54%), but only over ~32% between the 1990s and the 2000s (significant over ~26%). The most pronounced changes in GSFDs occurred in Eurasia, particularly in Europe and central Siberia (Fig. 2b, c), where the number of GFSDs increased between the 1980s and the 1990s, and decreased between the 1990s and the 2000s. The number of GSFDs increased most (>4.0 additional frost days per growing season, *P* < 0.05, *t*-test) in northern Europe between the 1980s and the 1990s and decreased most (>2.4 fewer frost days per growing season, *P* < 0.05, *t*-test) in central Siberia between the 1990s and the 2000s (Fig. 2b, c). These changes were more common in spring than in autumn over the last 30 years (Fig. 2), and the changes in SPR-FDs nearly mirrored the changes in GSFDs. Regions with consistent decreases in the number of GSFDs during the periods 1980s-1990s and 1990s-2000s are northeastern Siberia and consistent increases in southeastern Canada (Quebec, Sault Ste. Marie, and Moosonee) (Fig. 2b, c). This characterization of changes in GSFDs between successive decades is rather insensitive to the choice of the gridded temperature data sets (Supplementary Fig. 4) and meteorological station data (Supplementary Fig. 5).

We also calculated the number of GSFDs in Europe using in situ observations where leaf unfolding and leaf senescence were defined as the start and end of growing season (European phenology network, see Methods), and compared the results with satellite-based estimates within the same periods (Fig. 2 and Supplementary Fig. 6). We found that the number of GSFDs inferred from in situ records was always lower than those derived from the satellite observations (Supplementary Fig. 6), likely due to the different spatial scales of the satellite (0.5° in this study) and in situ (points) observations^25,26^. Good correlations were found between phenology data derived from GIMMS NDVI_3g.v1_ and MODIS EVI (SOS: R = 0.93, *P* < 0.01, *t*-test; EOS: R = 0.59, *P* < 0.01, *t*-test), suggesting the robustness of phenology-extraction methods across different satellite data sets. Satellite-derived phenology generally indicates a start of greening at a pixel scale, which is dominated by the signal of the earliest species (often ground cover), with EOS being dominated by the latest, and typically different, species in the corresponding pixel^27,28^. In contrast, in situ phenological data are always derived from individual trees, typically late-flushing species whose growing season is much shorter than that of the entire spectrum of species in the satellite image. Another possible reason was ascribed to the fact that satellite derived SOS and EOS might not tightly relate to the actual leaf unfolding and plant senescence^29,30^. Despite the difference in absolute numbers of GSFDs between in situ and satellite-based calculations, the temporal pattern of GSFDs based on in situ data is consistent with that of the satellite observations in Europe (Fig. 2 and Supplementary Fig. 6). The number of in situ based GSFDs increased between the 1980s and the 1990s, with averages of 0.6 ± 0.9, 0.6 ± 1.0, and 0.3 ± 0.7 additional GSFDs (991 sites), SPR-FDs (2655 sites) and FAL-FDs (1213 sites) per year, respectively (*P* < 0.05, *t*-test). The number of in situ based GSFDs declined significantly between the 1990s and the 2000s, similar to the satellite-based GSFDs, by 0.5 ± 1.0, 0.4 ± 1.0, and 0.1 ± 0.6 fewer GSFDs, SPR-FDs and FAL-FDs per year, respectively (*P* < 0.05, *t*-test) (Supplementary Fig. 6a). We found the same decadal changes of in situ GSFDs using other gridded-temperature data sets, namely CRU-NCEP, Princeton and WFDEI (Supplementary Fig. 6b–d).

### The contribution of phenology and temperature to GSFDs

Changes in plant phenology (GSL) and in *T*_min_ can both influence changes in GSFDs. To separate the contribution of these two factors, we simulated the number of growing season frost days based on our 30-yr observation record by (1) letting *T*_min_ change according to temperature observations and keeping phenology constant (scenario 1, see Methods for details), and (2) by letting phenology change as in satellite observations but keeping *T*_min_ constant (scenario 2, see Methods for details). The comparison in GSFDs between the two simulations and the actual estimates allows us to separate the contribution of changes in GSL vs. changes in *T*_min_. Changes in *T*_min_ alone lead us to observe a decrease in the number of GSFDs between the 1980s and the 2000s across most of the Northern Hemisphere (Supplementary Fig. 7a). In contrast, the simulation with changes in GSL alone produces an increase in the number of GSFDs (Supplementary Fig. 7b), by increasing the exposure of photosynthetically active vegetation to periods when *T*_min_ < 0 °C. Overall, change in *T*_min_ was found to have a larger effect in reducing GSFDs across most of Asia and North America in 2000s compared to 1980s (Supplementary Fig. 7a and c). On the contrary, the extension of GSL (primarily the advance of SOS) contributed more to the increase in the number of GSFDs in Europe (Supplementary Figs. 7c, e, f, 8a, d). Besides the spatial discrepancies in factors regulating the change in GSFDs, extended GSL was the dominant driving factor determining the increasing GSFDs between the 1980s and the 1990s, opposite to the extensively reduced GSFDs due to changes in *T*_min_ during the period 1990s and 2000s (Supplementary Fig. 7). This change in the driving factor of the GSFDs changes between the first two and the last two decades is mainly linked to spring rather than to autumn (Supplementary Fig. 7), probably because the advancing trend in SOS between the 1980s and the 1990s stalled or even reversed between the 1990s and the 2000s^19,20^. The same analysis based on three individual climatic data sets also produced similar results (Supplementary Fig. 7).

### Evidence for the hypothesis

**Fig. 3:**
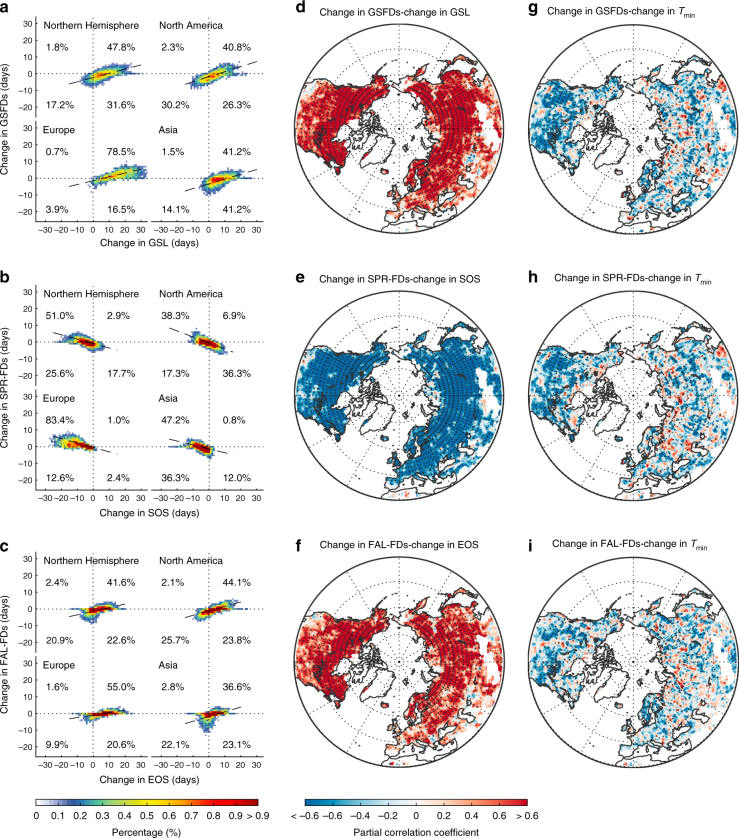
Relationship between the changes in phenology and corresponding frost days. This relationship is presented between the 1980s and the 2000s at both continental and local scales. The number of frost days was averaged from the results of the Princeton and WFDEI data sets. The colors in the left panels (**a**–**c** heating plots with percentage of pixels in each quadrant, continental scale) indicate the proportions of pixels that fall within each binned area (i.e., every 1 day in the changes of the number of frost days and phenology) in the Northern Hemisphere. The center (**d**–**f**) and right (**g**–**i**) panels display the spatial partial correlations between changes in the number of frost days with phenology and *T*_min_, respectively, using a moving window of 2.5 × 2.5° (i.e., local scale). Dotted regions indicate the correlations that were significant at *P* < 0.05, *t*-test. Maps were created using Matlab R2014b

We further tested the hypothesis that the number of GSFDs would increase as the growing season gets longer by exploring the spatial relationship between satellite derived phenological change and GSFDs in the Northern Hemisphere at both continental (Fig. 3 and Supplementary Fig. 9) and local (Fig. 3 and Supplementary Fig. 10) scales. At the continental scale, the number of GSFDs generally increased more in regions with faster extension of GSLs during all study periods (*P* < 0.01, *t*-test), despite the warming (Fig. 3a and Supplementary Fig. 9a–c). This association between increasing GSFDs and a longer GSL was more apparent in spring (Fig. 3b and Supplementary Fig. 9d–f) than autumn (Fig. 3c and Supplementary Fig. 9g-i). At the local scale, the spatial partial correlation with a moving window of 2.5 × 2.5° also revealed that regions with longer GSL tended to have more frost days during the growing season (Fig. 3 and Supplementary Fig. 10). More than 69% of the Northern Hemisphere shows a significant positive partial correlation between changes in GSL and changes in the number of GSFDs in each decade, after statistically removing the effect of temperature change (Fig. 3d and Supplementary Fig. 10a–c). This relationship was much stronger than the partial correlation between the changes in GSFDs and temperature (Fig. 3g and Supplementary Fig. 10a–c), indicating that the phenological change influenced the spatial pattern of changes in the occurrence of frost during growing season at the local scale more than the change in temperature. Analyses using the individual climatic data sets supported this result (Supplementary Figs. 9 and 10).

## Discussion

Our result shows that GSFDs increased mainly in Europe during the last three decades, indicating an increase in the vegetation exposure to cold events was found. However, it is still unclear whether more frost days during the growing season would result in more actual plant damage^31,32^. The susceptibility of plant growth to frost varies across species, and conditions^4,33^, which make it hard to directly compare the actual frost damage via accounting the number of frost days during growing season. Moreover, the susceptibility of plants to frost was found to increase with the specific growth stage^34^. Because the GSFDs are not equally distributed during the growing season (Supplementary Fig. 3a,b), the timing when the frost events occurred should not be neglected when assessing the impact of frost on plants.

In this study, we used in situ phenological observations (distributed in Europe and only deciduous tree species) to complement the results based on satellite-derived phenology. Although consistent changes in GSFDs were found, the large discrepancies between in situ- and satellite- based phenology dates (due to different spatial scales and inclusion of understory species in the satellite images) as well as in the reported changes in GSFDs, especially across other regions or species, should be investigated and validated in future in situ and experimental studies. Moreover, GSFDs predominantly occurred in the short periods after SOS and before EOS (Supplementary Fig. 3a,b), suggesting that the definition of SOS and EOS from NDVI data would impact the quantification of GSFDs. Such concern, however, was less obvious in estimating changes in GSFDs, because the result based on a single method (i.e., Piecewise logistic method, Supplementary Fig. 4) produced a similar pattern as the result based on the mean of four methods (Fig. 2). Previous studies largely focused on both in situ- and satellite- based spring phenology^15,28,35^, but payed less attention on autumn phenology^11,36^. This likely originates from the facts that autumn phenological events, such as leaf senescence, cannot be as easily assessed by abrupt visual signals as is the case for spring leaf out^30^, and the mechanisms underlying autumn phenology remain largely unknown^37^. The periods used for determining autumn frost days are less consistent as the periods used for spring frost days. In addition, the susceptibility of plant growth to frost during spring and autumn varies across species, locations and different growth stages^4,33,34^. Therefore, in situ observations and field experiments are urgently required to improve the understanding of the linkage between autumn phenology and autumn frost damage over the Northern Hemisphere.

In summary, the results of this study suggest that temperate and boreal vegetation ecosystems have been experiencing significant changes in the number of GSFDs. We found that the number of GSFDs generally increased with the lengthening of the growing season (especially in Europe and in spring) but decreased in some regions due to global warming during the last three decades. Moreover, GSFDs were less frequent in 2000s than 1990s, mainly because the SOS stopped advancing or even came later in 2000s during the warming hiatus periods. The impact of frost occurrence during growing season is likely species-specific and also the carry-over effect might be different among species. It was suggested that frost damage influences the timing of leaf-out in temperate tree species^38^ and damages flower buds and seeds of montane wildflowers^3^, reduces the gross productivity of forest ecosystem^5^ and decreases the yield of economical crops^9^. The increase in frost occurrence during growing season could modulate the magnitude and even direction of the response of regional vegetation growth to climate change^1,39,40^ and may offset some of the benefits of a longer growing season, such as the enhanced productivity in northern ecosystems. A longer growing season may be a major mechanism for increasing productivity in the Northern Hemisphere under global warming^41–44^. Most state-of-the-art models of the Earth ecosystem, however, do not take into account the impacts of increasing growing season frost occurrence on vegetation growth, implying that the ability of northern ecosystems to sequester carbon may be overestimated. Acquiring a better understanding of growing season frost occurrence and its potentially damaging impact on vegetation productivity is clearly a priority for developing strategies to reduce the vulnerability of ecosystems under future climate change.

## Methods

### Global climatic data sets

We extracted data for daily minimum temperature (*T*_min_) for calculating the number of GSFDs from three independent global climatic data sets and one station-level data set from Global Surface Summary of the Day (GSOD). Three gridded data sets provided globally continuous records with a spatial resolution of 0.5 × 0.5°, but each was sampled with a different time interval for 1982 to 2012. First, CRU-NCEP v5 (CRU: Climatic Research Unit, NCEP: National Centers for Environmental Protection, hereafter CRU-NCEP, ftp://nacp.ornl.gov/synthesis/2009/frescati/model_driver/cru_ncep/analysis/readme.htm) is a 6-h data set based on the combination of records from globally distributed terrestrial meteorological stations and NCEP reanalysis data^45,46^. Second, we used a 3-h sampled global data set produced by the Terrestrial Hydrology Research Group at Princeton University^47^ (hereafter Princeton, available from http://hydrology.princeton.edu/data.pgf.php). This data set merges a reanalysis^48^ with global observations and is designed for modeling hydrological and land-surface processes. Temperature in this data set was corrected to match the Climatic Research Unit (CRU) time series (TS) v3.0 data set on a monthly scale before data publication^46^. Last, we used the WFDEI meteorological forcing data set (WATCH Forcing Data methodology applied to ERA-Interim data, http://www.eu-watch.org/data_availability), which uses data from an ERA-Interim reanalysis and provides *T*_min_ at time steps of 3 h^49^. All three data sets have been successfully applied in recent studies of climate change^47,50^. GSOD data set was released by the National Climatic Data Center (https://data.noaa.gov/dataset/global-surface-summary-of-the-day-gsod). We extracted 2626 stations with 31 years of available minimum temperature over the study period 1982–2012, and these stations were distributed across most of our study area.

### Satellite-derived phenology

The seasonal variations of the Normalized Difference Vegetation Index (NDVI), a proxy of vegetation greenness and photosynthetic activity^51^, is commonly used to interpret phenometrics (e.g., growing-season length (GSL), start of the growing season (SOS), and end of the growing season (EOS))^52^. We derived the phenology in the Northern Hemisphere from the latest generation of NDVI records by NASA’s GIMMS group (GIMMS_3g_._V1_, an updated from previous GIMMS_3g_). Errors and noise associated with the update of the satellite sensors, atmospheric interference, and non-vegetation dynamics were addressed in GIMMS_3g_, while artifacts due to changes in calibration and replacement of negative NDVI in snow-covered regions with zero values in previous version have been further processed^53,54^. We applied four widely used methods to extract phenology dates: HANTS-Mr^55^, Polyfit-Mr^56^, Double logistic^57^ and Piecewise logistic^58^. For the HANTS-Mr and Polyfit-Mr methods, we fitted NDVI time-series via “harmonic analysis”^55^ and “six-order polynomial function”^56^ and determined the phenology date with maximum increase/decrease in NDVI. For the Double logistic method, we used a double logistic function to smooth the NDVI data and the date of SOS/EOS was embedded in the formulation of this function (i.e., a model parameter)^57^. The Piecewise logistic method used pairs of sigmoid function to fit the seasonal NDVI curve and defined the local maxima/minima for the derivatives of fitted NDVI curve as SOS/EOS^58^. We firstly fitted the 24 bimonthly composited NDVI data averaged during 1982–2012 to remove the noise and then the day with maximum decrease in NDVI (differed in its definition in each method) during the second half of the year was identified as EOS^59^, while the day with maximum increase in NDVI during the first half year was identified as SOS. Then, we used the NDVI of these dates as thresholds to estimate SOS and EOS in individual years. For pixels with two complete growing seasons, we used the start of the first growing season and end of the second growing season as the SOS and EOS of the entire year. However, for pixels with two growing seasons, but with the second growing season ending in the next year, we only took the first growing season into consideration. The combined mean from four methods was used for determining the growing season for each pixel at each year to minimize the uncertainty associated with their discrepancies in interpreting phenological information from the NDVI seasonal curve. Moreover, we compared NDVI-based phenology data with EVI-based phenology data to complement our analysis. Limited by the time span of MODIS EVI dataset (since 2000), we only compared the SOS/EOS dates over the period 2000–2009. To eliminate the spatial mismatch between these satellite data sets (i.e., 1/12 × 1/12° in GIMMS NDVI_3g.v1_ and 0.05 × 0.05° in MODIS EVI), we remapped the extracted phenology data into the same spatial resolution (0.5 × 0.5°).

### In situ phenological data

In situ phenological data were downloaded from the PAN European Phenology network (project PEP725, http://www.pep725.eu/index.php), which offers open and unrestricted access to long-term phenological records from 26 European countries. The dates of leaf unfolding (i.e., first visible leaf stalk) and leaf senescence (i.e., 50% of autumnal coloring) were inferred from the ancillary BBCH code (Biologische Bundesanstalt, Bundessortenamt und Chemische Industrie). To avoid potential bias due to outliers and insufficient observations, we did not use sites with leaf unfolding later than the end of June and leaf senescence earlier than the beginning of July and concentrated on sites with 28 years of observations of leaf unfolding (2655), leaf senescence (1213), and both (991) for 1982–2009.

### Calculation of the number of frost days

Frost days were defined as days when *T*_min_ was below freezing point of water (0 °C)^17,18^. We remapped the satellite-derived phenological data (1/12° spatial resolution) to spatially match the spatial resolution of the climatic data set (*T*_min_, 0.5° spatial resolution). First, we calculated the number of frost days during the growing season (GSFDs, from SOS to EOS) in the Northern Hemisphere for 1982–2012. We then divided our calculations into spring (SPR-FDs, from SOS to the summer solstice (i.e., 22 June in the Northern Hemisphere)) and autumn (FAL-FDs, from the summer solstice to EOS). We calculated the number of GSFDs under two scenarios to further distinguish between the effects of phenological and temperature trends on the change in the number of GSFDs in the Northern Hemisphere during the last three decades. In the first scenario, we only considered temperature changes and kept phenology constant (i.e., the phenological data were randomly selected for 1982–2012 and held constant). In the second scenario, we varied only phenology and used a constant temperature (i.e., temperature data were randomly selected for 1982–2012 and held constant). We randomly selected phenology or *T*_min_ 10 times for 1982–2012, applied them individually in the estimations, and used their averages to eliminate sampling bias. Due to the difference in temporal resolution, we presented the average number of GSFDs from the results of the 3-h Princeton and WFDEI data sets and listed the results inferred from the three individual data sets as alternative choices in the Supplementary Figs. 2–10. We also calculated the GSFDs from in situ observed leaf unfolding to leaf senescence using three gridded climatic data sets, then separated them into SPR-FDs (from leaf unfolding to summer solstice) and FAL-FDs (from summer solstice to leaf senescence). Moreover, station-level GSOD minimum temperature was applied to replace gridded climatic data sets to complement our result. Finally, we also examined the robustness of using summer solstice as the cut-off point to separate GSFDs into SPR-FDs and FAL-FDs through two ways: (1) calculating the temporal distribution of SPR-FDs and FAL-FDs within each 10 days’ periods after SOS and before EOS, individually. (2) using the middle day between SOS and EOS to test whether the choice of summer solstice as the cut-off point of growing season would impact the contribution of SPR-FDs and FALL-FDs to GSFDs.

### Data availability

The authors declare that the source data supporting the findings of this study are provided with the paper.

## Electronic supplementary material

Supplementary Information
