# Supplementary material for: Extension of the growing season increases vegetation exposure to frost
Source: Nat Commun. 2018 Jan 30;9:426. doi: 10.1038/s41467-017-02690-y (PMC5789858; doi:10.1038/s41467-017-02690-y)
Supplement: Supplementary file 1 — Supplementary Information [file 41467_2017_2690_MOESM1_ESM.pdf]

Supplementary Figure 1. Spatial distribution of satellite-derived phenology. Growing season length (GSL, **a**), start of growing season (SOS, **b**), end of growing season (EOS, **c**) averaged during the period 1982-2012. SS in the color bar indicates the date of summer solstice.

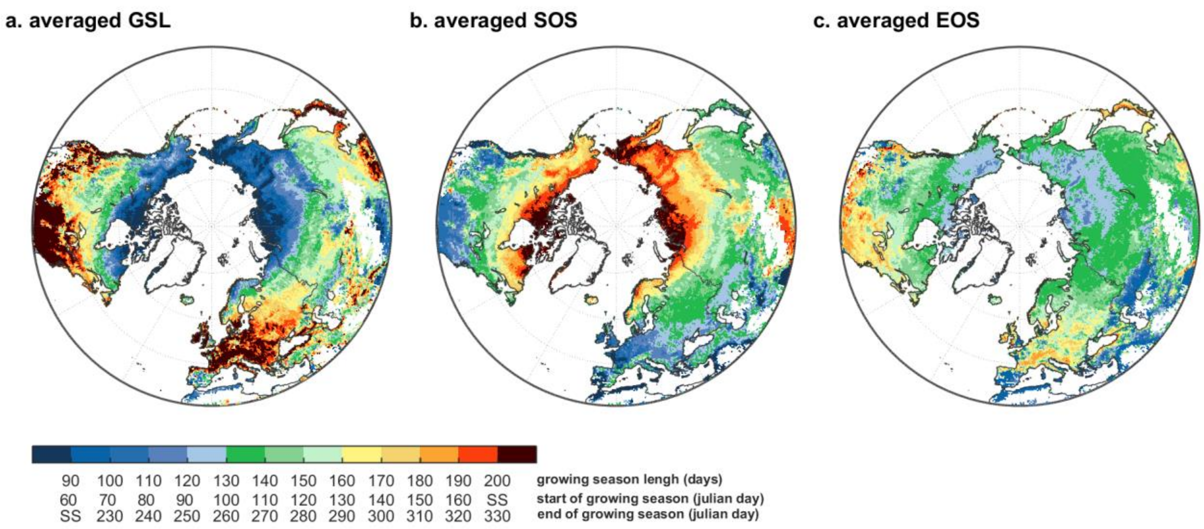

Supplementary Figure 2. Spatial distribution of average frost days during growing season for 1982-2012. **a-d**, the number of frost days averaged from the results of the Princeton and WFDEI datasets. **a-c** indicates frost days and their variation along the gradient of latitude (black line and grey area presents the average frost days and its standard deviation across latitudes) calculated during growing season (GSFDs, from SOS to EOS), spring (SPR-FDs, from SOS to the middle day between SOS and EOS) and autumn (FAL-FDs, from the middle day between SOS and EOS to EOS). **d** displays the ratio of the number of frost days between **b** and **a**. **e-p** delivered similar results based on CRU-NCEP, Princeton and WFDEI datasets, while summer solstice is applied to separate the growing season into spring (SPR-FDs, from SOS to the summer solstice) and autumn (FAL-FDs, from the summer solstice to EOS).

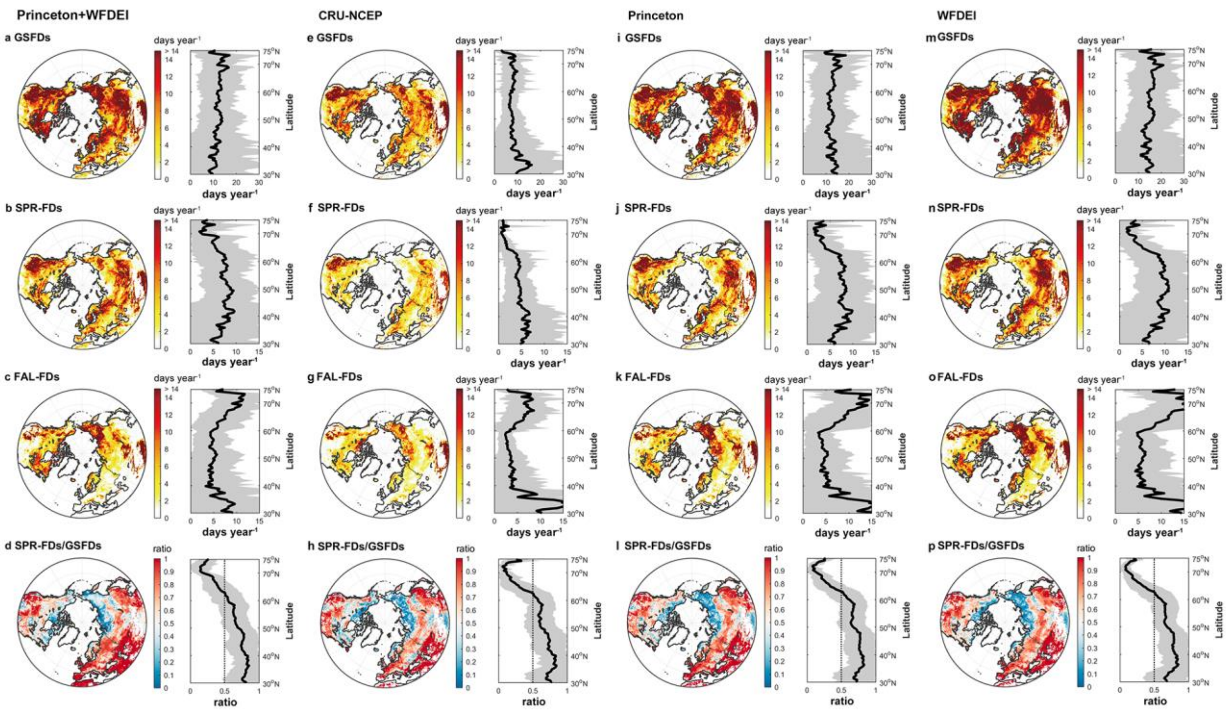

Supplementary Figure 3. Temporal distribution of growing season frost days. **a** and **b** shows the percentage of frost days within 10 days' period towards the start and end of growing season during 1982-2012. Bars and error bars suggest the mean ± s.d. percent of frost days occurred in each 10 days' period after SOS (**a**) and before EOS (**b**). The circles represent the cumulative percentage of frost days in spring (**a**) and autumn (**b**). **c** and **d** shows the spatial pattern of increased spring frost days (SPR-FDs) between the periods 1982-1989 and 2000-2009, and its distribution with 10 days' period after the start of growing season (SOS), respectively. Similarly, **e** and **f** presents the distribution of increased SPR-FDs between 1982-1989 and 1990-1999.

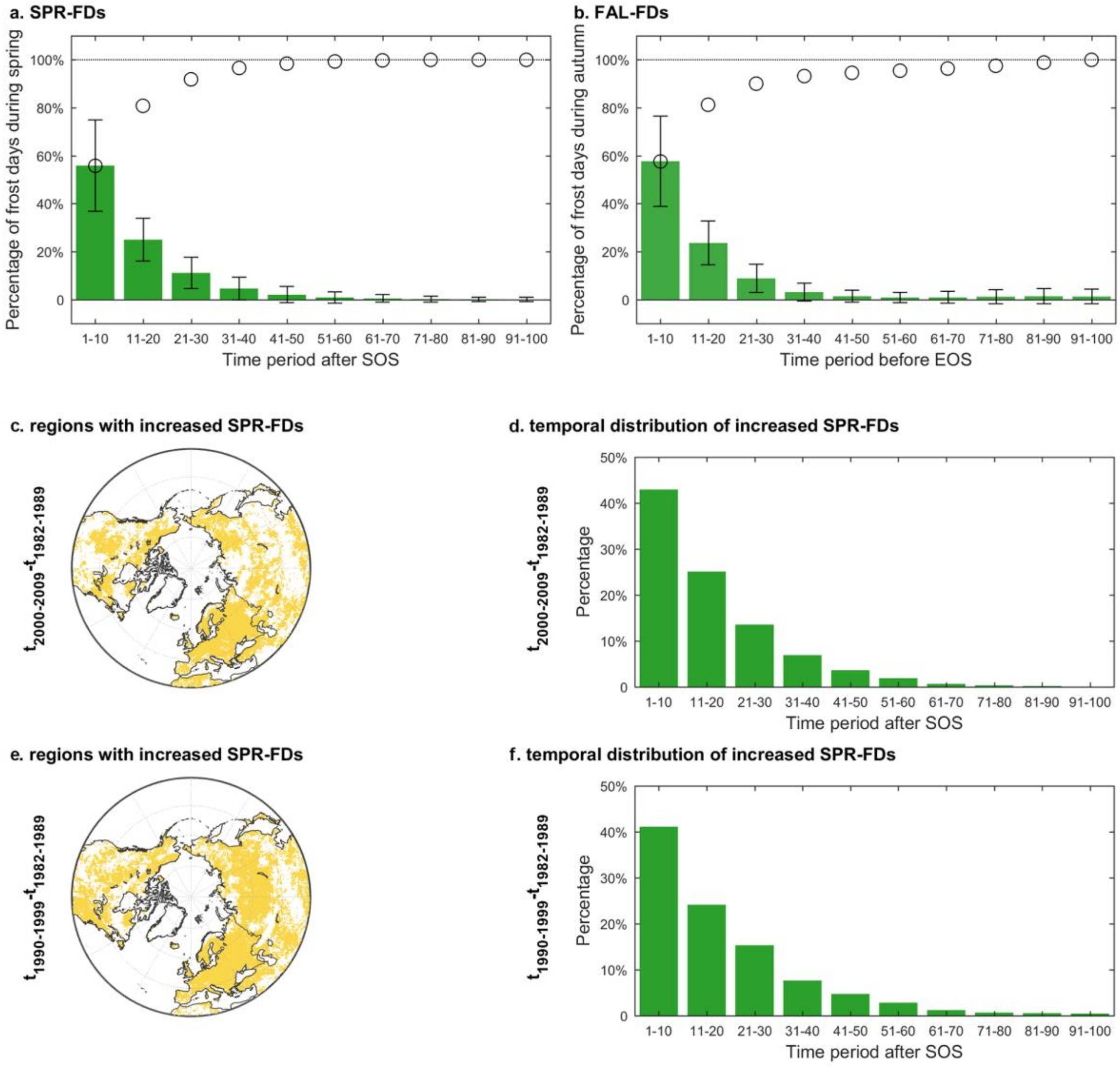

Supplementary Figure 4. Decadal changes in average frost days during growing season. The number of frost days (FD) in the Northern Hemisphere was averaged during the 1980s-2000s, 1980s-1990s, and 1990s-2000s. The upper left panels (**a-i**), upper right panels (**j-r**), and bottom left panels (**s-a1**) indicate the change in the number of frost days (FD) during the growing season (GSFDs, from SOS to EOS), spring (SPR-FDs, from SOS to the summer solstice), and autumn (FAL-FDs, from the summer solstice to EOS) derived from the CRU-NCEP, Princeton, and WFDEI datasets, respectively. While, the bottom right panels (**b1-j1**) are the result based on phenology derived from double logistic method and Princeton, WFDEI datasets. Dotted areas indicate regions with significant changes in the number of frost days at $P < 0.05$ (t-test).

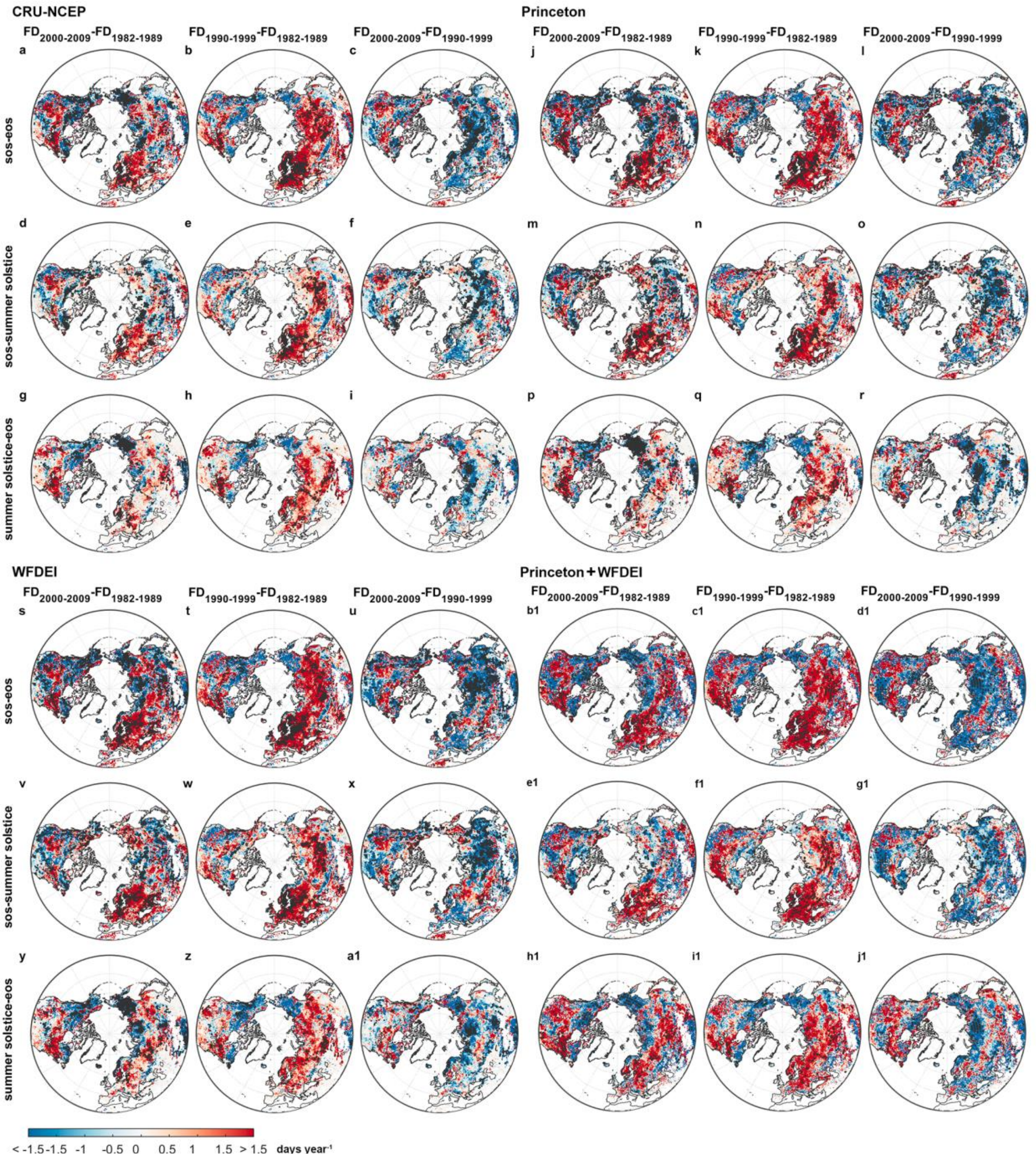

Supplementary Figure 5. Decadal changes in average frost days during growing season. The number of frost days (FD) in the Northern Hemisphere was averaged during the 1980s-2000s, 1980s-1990s, and 1990s-2000s. FD is calculated based on satellite-derived phenology data and the station-level T_{min} from Global Surface Summary of the Day (GSOD) dataset. The left, center, and right panels indicate the differences in the average number of frost days on a decadal scale. The upper, middle, and bottom panels show the time periods used to calculate the number of frost days (**a-c**: GSFDs, from SOS to EOS, **d-f**: SPR-FDs, from SOS to the summer solstice, and **g-i**: FAL-FDs, from the summer solstice to EOS).

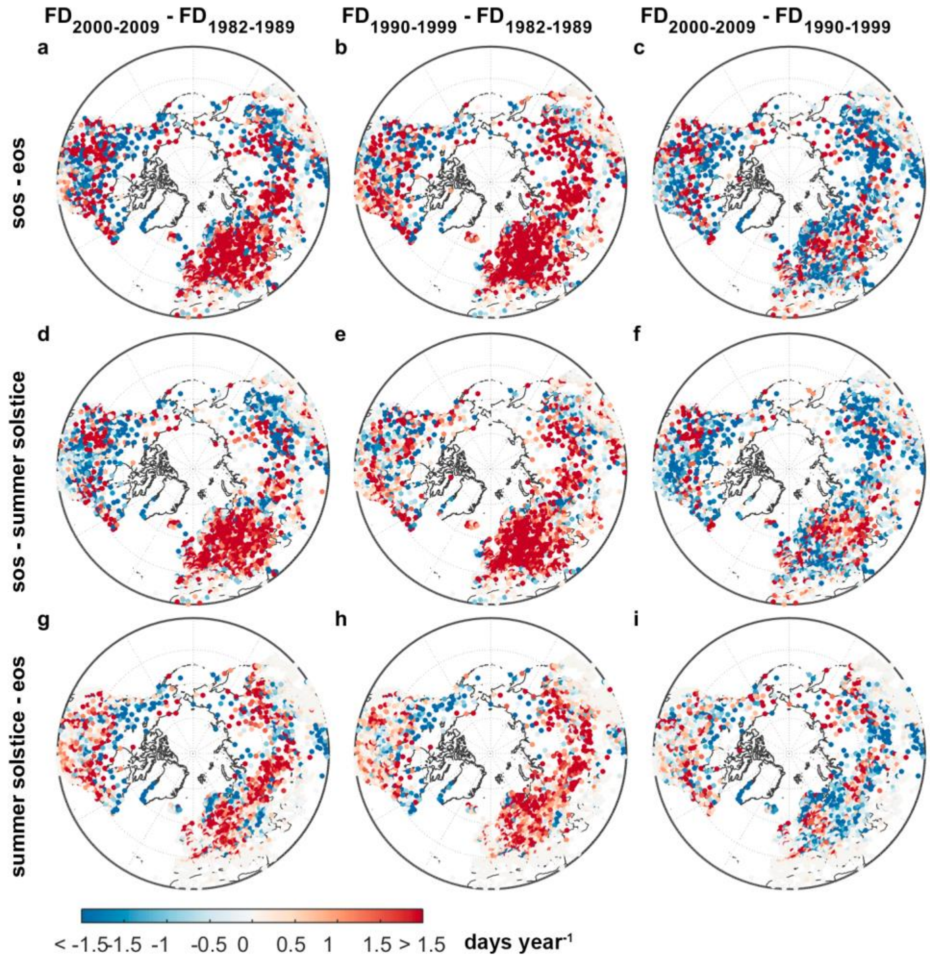

Supplementary Figure 6. Histogram of the changes in frost days during the past decades. Frost days (FD) is calculated based on satellite and *in situ* observations in Europe of phenology. **a**, frost days averaged from the results of the Princeton and WFDEI datasets (upper left panels). **b**, **c**, **d**, frost days calculated using CRU-NCEP, Princeton, and WFDEI datasets (the remaining panels), individually. * indicates that changes in the number of frost days were significantly different from 0 (t-test, P < 0.05).

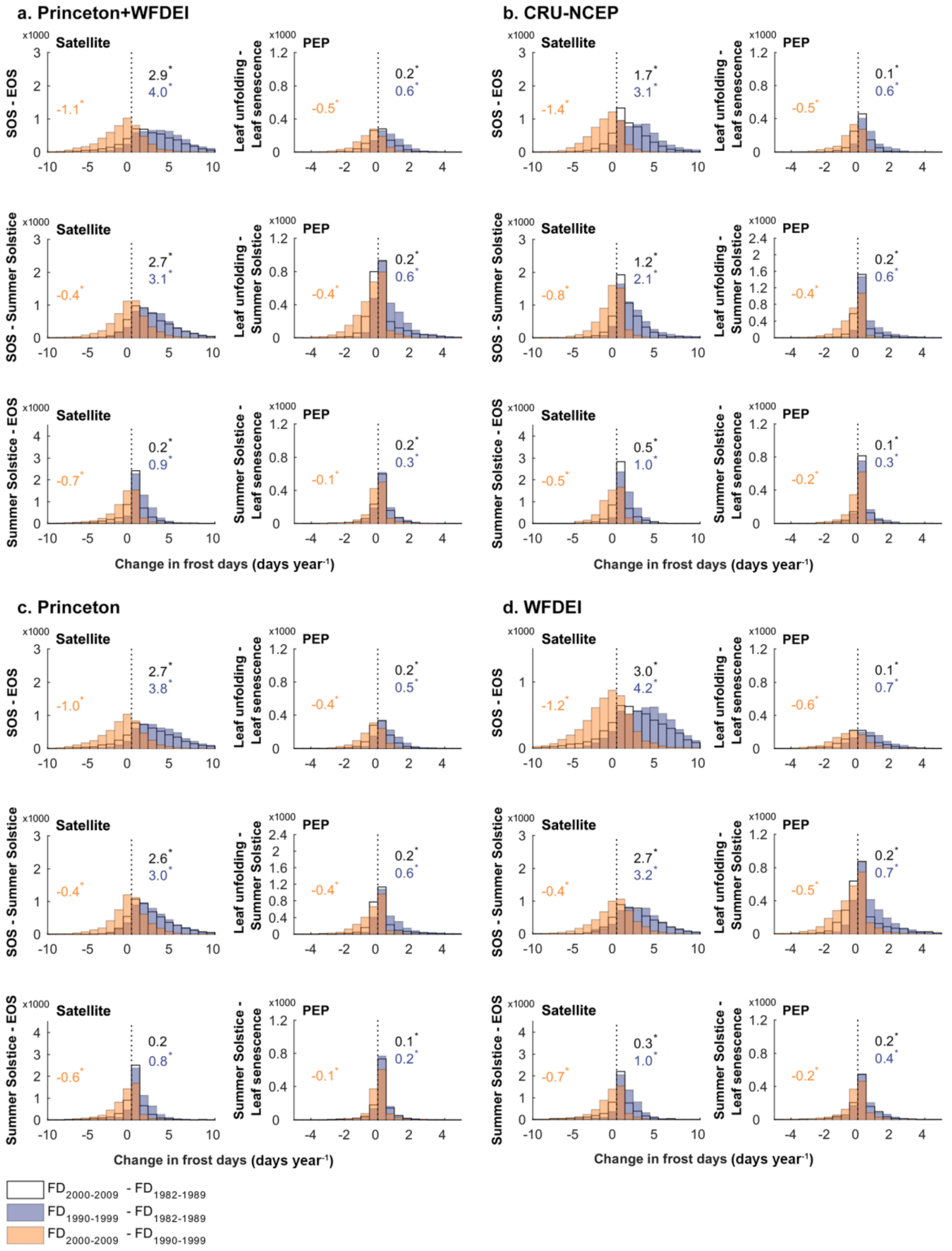

Supplementary Figure 7. Spatial pattern of changes in frost days under two scenarios. Scenario 1 (varying temperature and keeping phenology constant) and Scenario 2 (varying phenology and keeping temperature constant) were designed to explore the effects of changes in phenology and climate on changes in the number of frost days (FD) across the Northern Hemisphere. The upper left panels (**a-i**), upper right panels (**j-r**), and bottom left panels (**s-a1**) indicate changes in the number of frost days (averaged from the results based on Princeton and WFDEI datasets) during the 1980s-2000s, 1980s-1990s, and 1990s-2000s. Dotted regions show where the sign of changes in the number of frost days were consistently observed in the CRU-NCEP, Princeton, and WFDEI datasets.

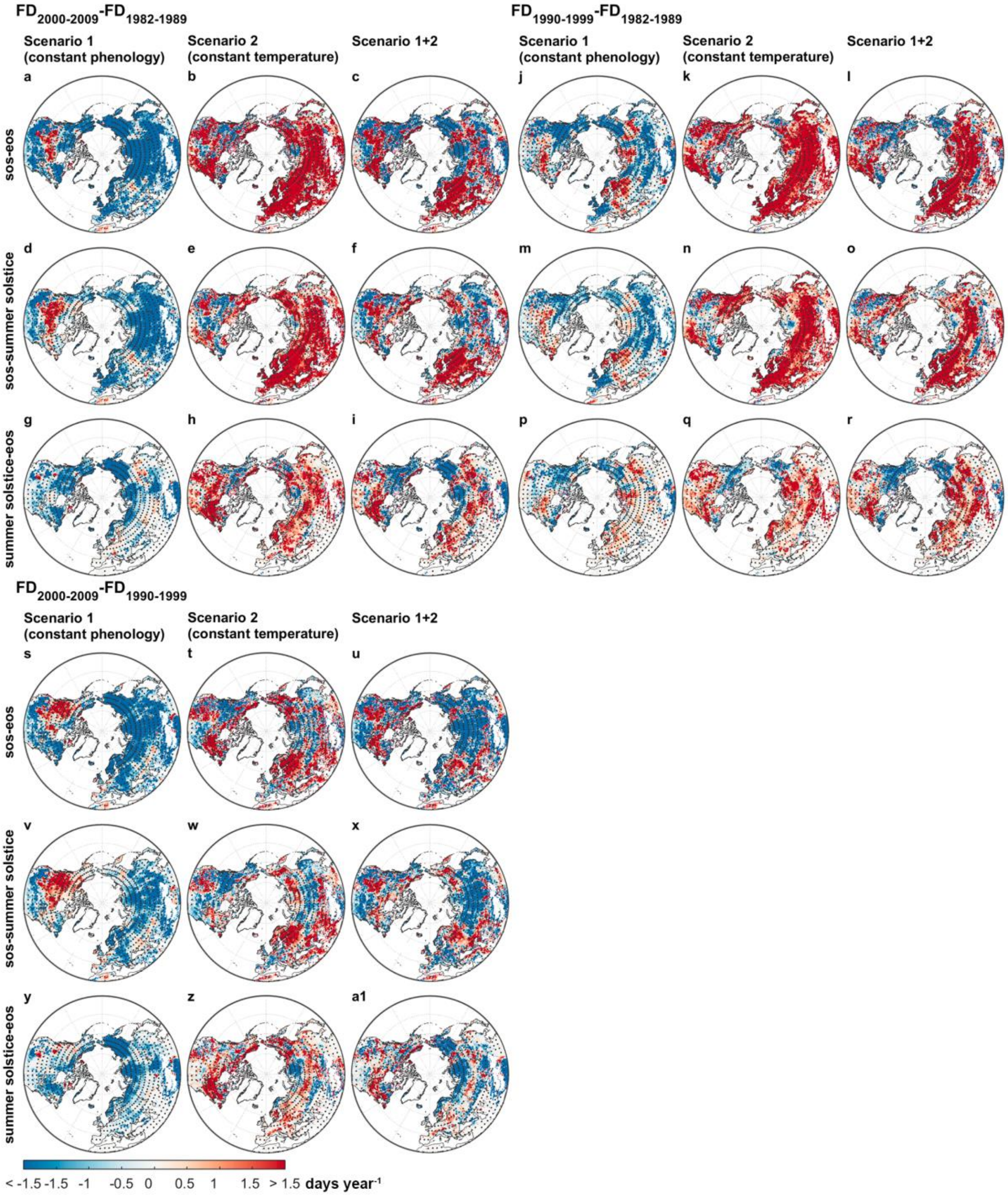

Supplementary Figure 8. Joint spatial pattern of changes in phenology and frost days. The upper left panels (**a-i**) display the number of frost days averaged from the results based on the Princeton and WFDEI datasets during the past three decades, while the upper right (**j-r**), bottom left (**s-a1**), and bottom right (**b1-j1**) show the number of frost days calculated individually from the CRU-NCEP, Princeton, and WFDEI datasets, respectively. Colors indicate the magnitude of changes in phenology (from red/yellow (negative) to green/blue (positive)) and frost days (from red/green (negative) to yellow/blue (positive)), respectively.

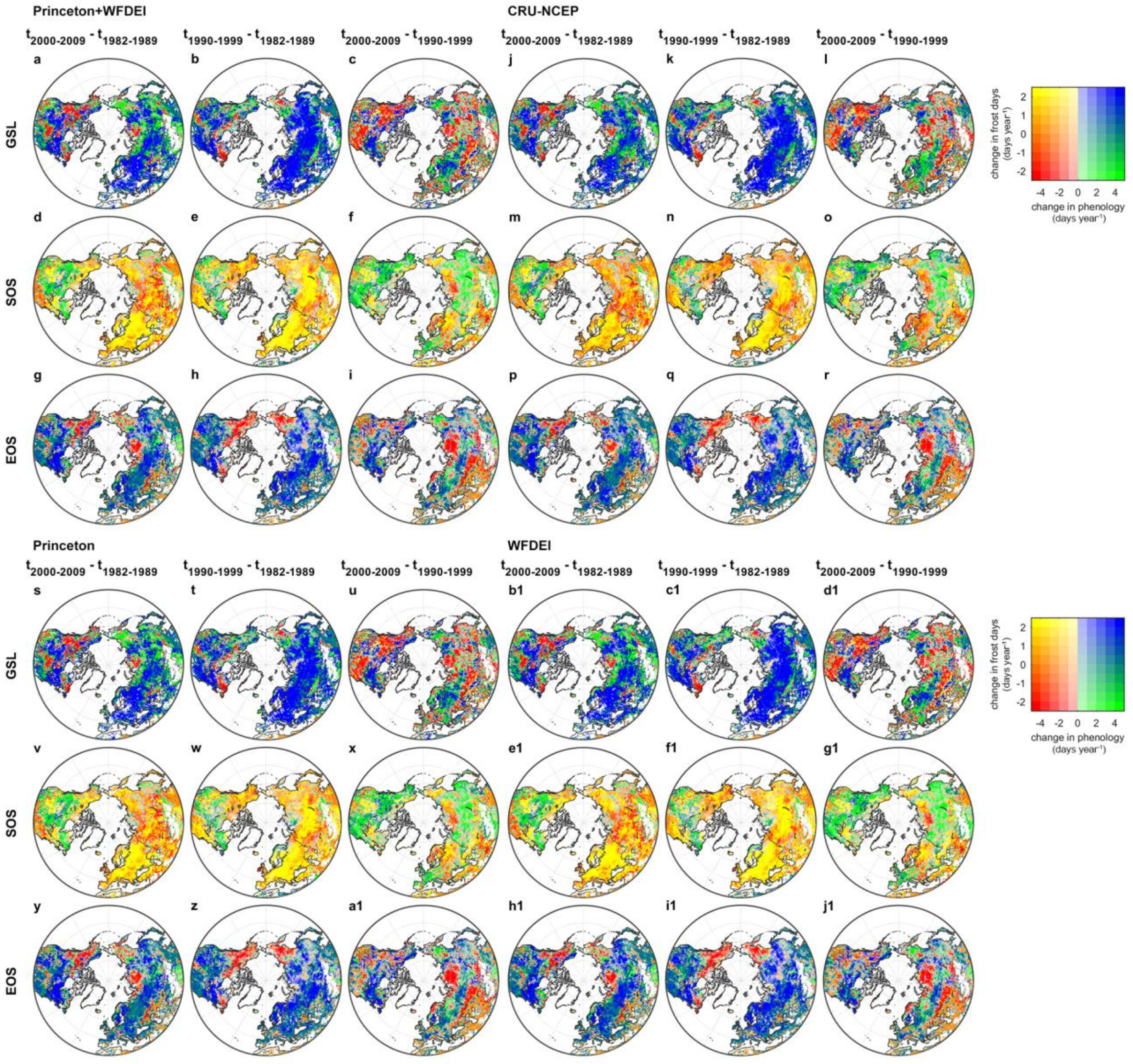

Supplementary Figure 9. Heat map showing the spatial relationship between changes in phenology and frost days. Upper left panels (**a-i**) represent the number of frost days averaged from the results based on the Princeton and WFDEI datasets across the Northern Hemisphere, while the upper right (**j-r**), bottom left (**s-a1**), and bottom (**b1-j1**) right panels show the number of frost days calculated individually from the CRU-NCEP, Princeton, and WFDEI datasets, respectively. Colors indicate the proportions of pixels that fall within each binned area (i.e. every one day in changes of frost days and phenology).

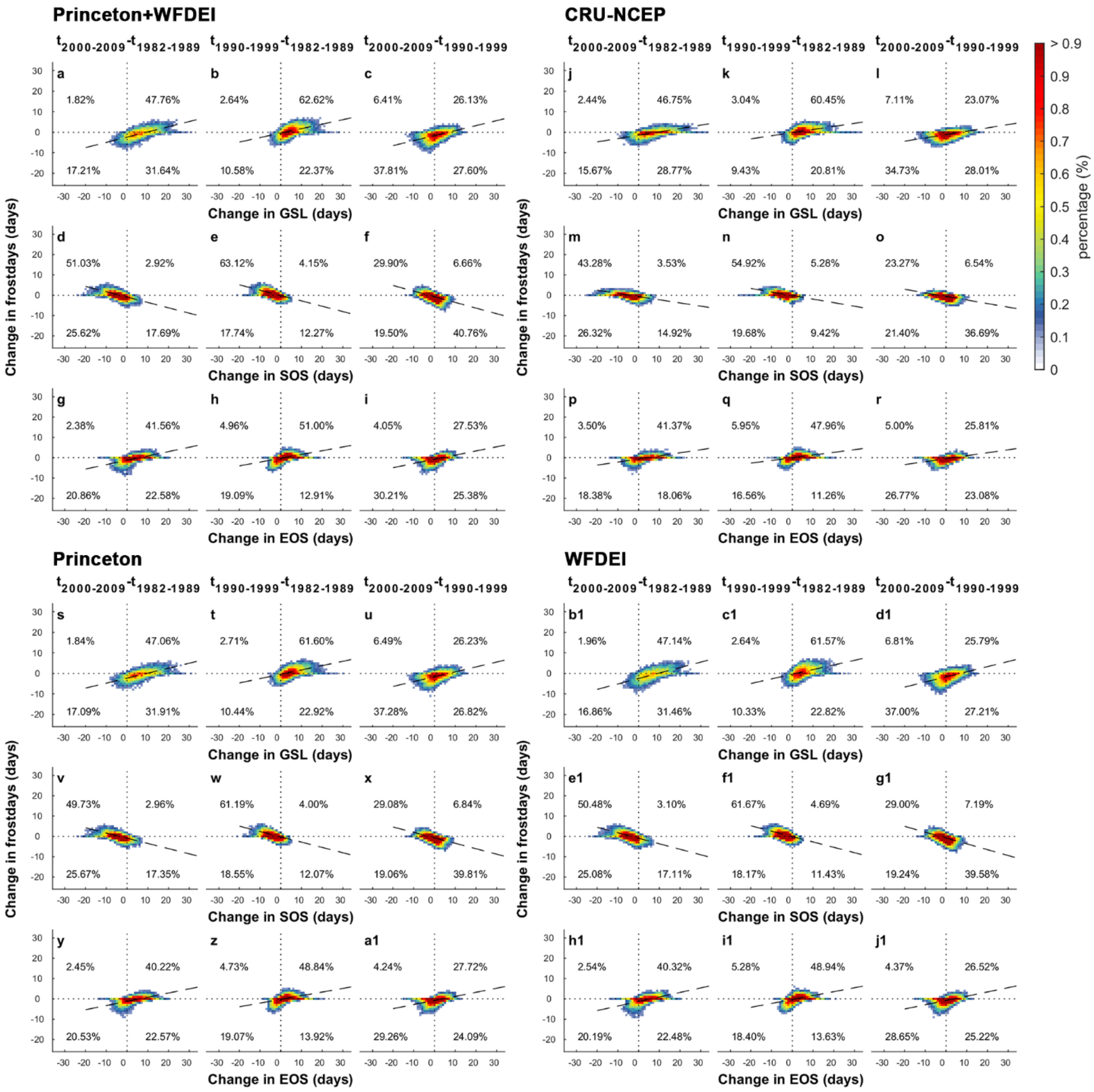

Supplementary Figure 10. Spatial partial correlations between changes in frost days, phenology, and temperature. Frost days (FD) and average Tmin were calculated during the periods from growing season (from SOS to EOS), spring (from SOS to the summer solstice), and autumn (from the summer solstice to EOS) using the combination of the Princeton and WFDEI (**a-i**), CRU-NCEP (**j-r**), Princeton (**s- a1**), and WFDEI (**b1-j1**) datasets. Spatial partial correlation was conducted over a moving window of 2.5° by 2.5° (local scale). Colors indicate the partial correlation coefficients ranging from negative (blue) to positive (red). Dotted areas suggest significant correlations at $P < 0.05$.

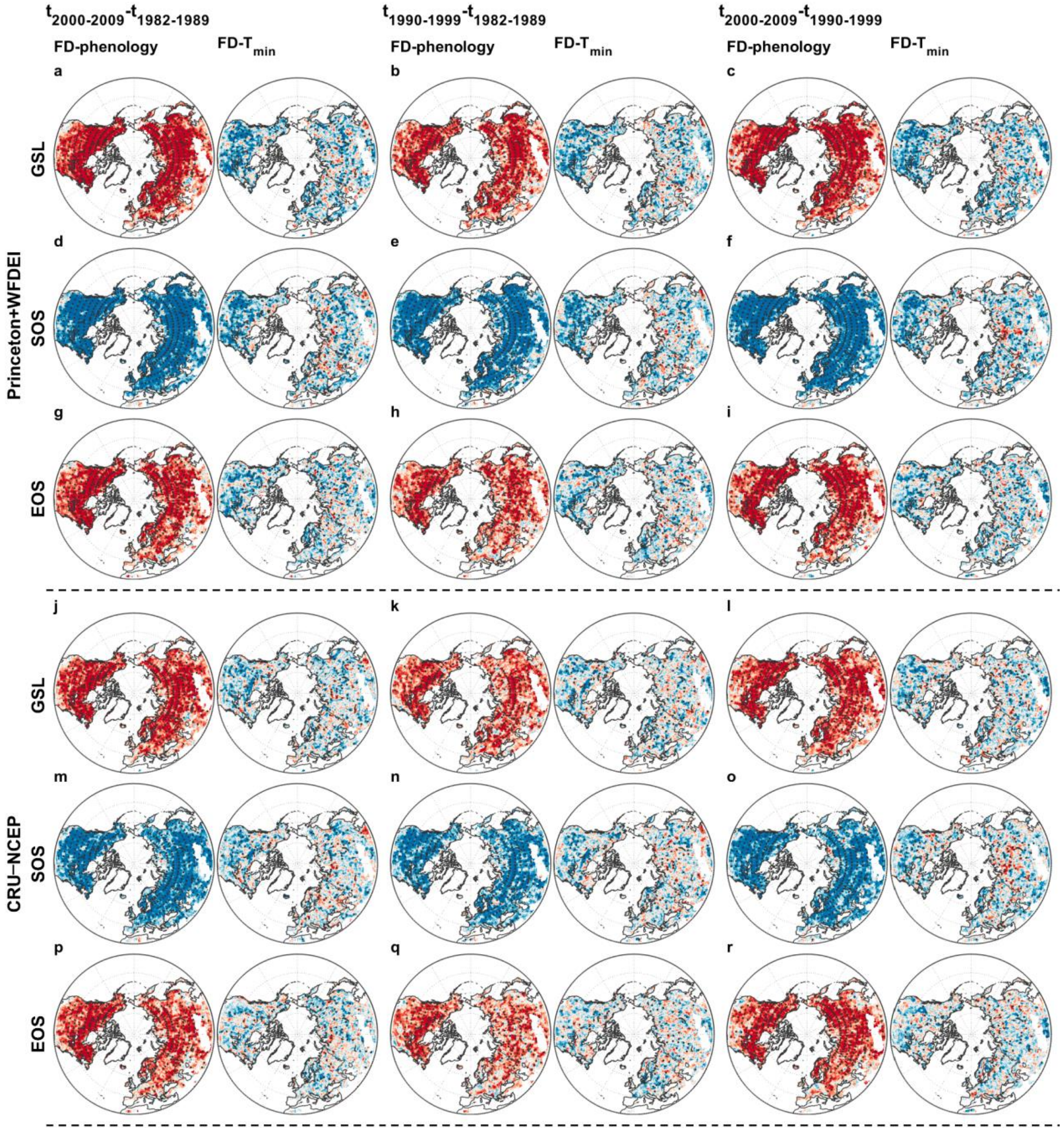

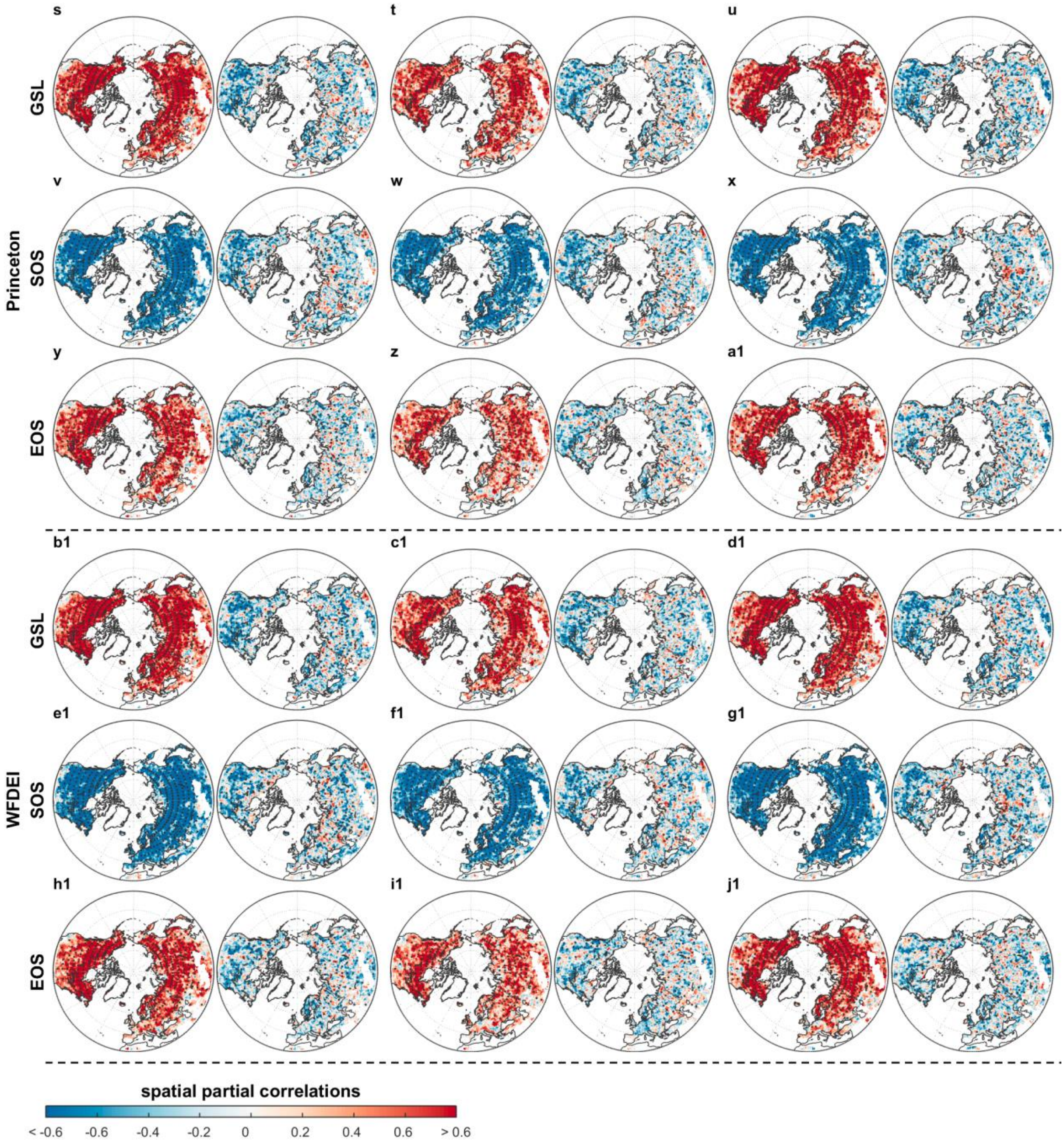
s
t
u
v
w
x
y
z
a1
b1
c1
d1
e1
f1
g1
h1
i1
j1
Princeton
WFDEI
GSL
SOS
EOS
GSL
SOS
EOS
spatial partial correlations
< -0.6
-0.6
-0.4
-0.2
0
0.2
0.4
0.6
> 0.6
